# Exogenous Melatonin Delays Dark-Induced Grape Leaf Senescence by Regulation of Antioxidant System and Senescence Associated Genes (SAGs)

**DOI:** 10.3390/plants8100366

**Published:** 2019-09-23

**Authors:** Xingyun Shi, Shanshan Xu, Desheng Mu, Ehsan Sadeghnezhad, Qiang Li, Zonghuan Ma, Lianxin Zhao, Qinde Zhang, Lixin Wang

**Affiliations:** 1 Wuwei Academy of Forestry Science, Wuwei 733000, China; shixingyunlove@163.com (X.S.); sfy1003@163.com (Q.Z.); lq870817@126.com (Q.L.); zlx.08.happy@163.com (L.Z.); zqd8559@163.com (Q.Z.); 2 College of Horticulture, Hebei Agricultural University, Baoding 071001, China; 3 Economic Crops Technical Advising Station of Huzhou City, Huzhou 313000, China; 4 Wuwei Management Office for Forestry and Fruit Industry, Wuwei 733000, China; 0603xss@163.com; 5 Department of Plant Biology, Faculty of Biological Sciences, Tarbiat Modares University, Teheran 14115-111, Iran; ehsansadeghnejad@yahoo.com; 6 College of Horticulture, Gansu Agricultural University, Lanzhou 730070, China; mazohu@163.com

**Keywords:** antioxidant enzymes, grape, leaf senescence, melatonin, reactive oxygen species, ascorbate-glutathione, leaf senescence-associated genes

## Abstract

Leaf senescence is a developmentally programmed and degenerative process which comprises the last stage of the life cycle of leaves. In order to understand the melatonin effect on grapevine leaf senescence, the dark treatment on detached leaves of *Vitis vinifera* L. cv. Red Globe was performed to induce leaf senescence at short period of time. Then, a series of physiological and molecular changes in response to exogenous melatonin were measured. Results showed that 100 μM of melatonin treatment could significantly delay the dark induced leaf senescence, which is accompanied by the decreased production of reactive oxygen species (ROS). Meanwhile, melatonin treatment could increase the scavenging activity of antioxidant enzymes, such as peroxidase (POD), superoxide dismutase (SOD), and catalase (CAT). Simultaneously, ascorbate (AsA) and glutathione (GSH) contents, the activities of ascorbate peroxidase (APX), and glutathione reductase (GR) were significantly higher than control treatment in samples treated with melatonin. Furthermore, melatonin treatment showed to suppress the expression of leaf senescence-associated genes (SAGs). All these results demonstrated that melatonin could activate the antioxidant and Ascorbate-Glutathione (AsA-GSH) cycle system and repress the expression of SAGs that lead to delay the dark induced grape leaf senescence.

## 1. Introduction

Leaf development plays vital roles in the growth and development of plant, especially when photosynthesis takes place in this main area [1]. The environmental conditions such as insufficient light, low CO_2_ concentration, high temperature, and humidity could affect the life cycle of leaves. For grapevines, these stress factors could not only influence their growth, but also accelerate the leaf senescence and affect the rate of photosynthesis that resulted in a reduction of yield and fruit quality [2,3]. Therefore, delaying leaf senescence of grapevines could be of vital importance to increase the level of fruit quality and production.

Leaf senescence, which is often considered as a type of programmed cell death, is the ultimate period of leaf growth in the vegetative cycle [4,5,6]. During this process, a series of physiological and biochemical events take place. For example, the most evident sign of leaf senescence is the color change from green to yellow through the degradation of chlorophyll, which lead to the decline of photosynthetic capacity [7,8]. Meanwhile, the degradation of macromolecular substances such as proteins and nucleic acids, oxidative degradation of lipids, and remobilization of nutrients influence the storage organ development [9,10,11].

In plants, leaf senescence process is regulated by developmental and various endogenous and exogenous environmental factors. The environmental factors as biotic or abiotic stresses mainly include darkness [12,13], detachment [14,15], water stress [16,17], salt or alkali stress [18,19], temperature stress [20,21], heavy metals [22,23], inappropriate light [24,25], nutrient deficiency [26,27] and pathogen infection [28,29]. The developmental factors consist of leaf age [30,31], plant hormones [32,33], senescence related genes [34,35] such as SAG12 and transcription factors [12,16,36], epigenetic regulation [37], etc. The regulatory mechanisms of senescence are interrelated and highly influenced by environmental factors. In addition, plant hormones also play crucial roles in leaf senescence. For example, ethylene (Eth) [38], abscisic acid (ABA) [39], salicylic acid (SA) [40] and jasmonic acid (JA) [41] could promote leaf senescence, while gibberellin (GA), cytokinin (CKs) and auxin can inhibit leaf senescence process [42,43,44,45]. Therefore, the mechanisms of leaf senescence are very complicated and deserve to be explored.

Melatonin (MT), known as N-acetyl-5-methoxytryptamine, is an endogenous indoleamine derived from the plant primary metabolism by decarboxylation of the amino acid tryptophan [46]. MT content in plants is very low [47], but it has increasingly vital functions, such as regulating growth of shoots and roots [48], seed germination [49], fruit ripening [50,51], and response to stresses [52,53,54,55] in the course of plant life. Additionally, some reports have demonstrated that MT plays a significant role in delaying leaf senescence during abiotic stresses such as heat, drought, darkness, salinity and cold [56,57,58]. Reactive oxygen species (ROS), mainly including O_2_^−^ and H_2_O_2_, are by products of aerobic metabolism in plants. Excess accumulation of ROS can accelerate a series of events involved in senescence [59,60,61]. Many studies have ascertained that MT as an antioxidant could directly decrease the ROS via clearing away free radicals. In addition, it could influence photosynthetic activity and the activity of antioxidant enzymes, such as peroxidase (POD), superoxide dismutase (SOD), catalase (CAT), and ascorbate peroxidase (APX) [55,56]. MT also increases the content of antioxidant defense agents such as ascorbate (AsA), glutathione (GSH), and flavonoids, which could protect antioxidant enzymes from oxidative damage and activate the senescence-associated genes (SAGs) to prevent or delay the damage in plants. The typical studies have been performed on kiwi, apple, rice and *Arabidopsis* [57,62,63,64]. However, how MT regulates the leaf senescence of grapevine has not been systematically elucidated and whether the same mechanism existed in grapevine is worth being detected.

The aim of this study was to illuminate the regulating mechanisms of MT on delaying leaf senescence in dark condition. The detached leaves of grapevine ‘Red Globe’ were used as experimental materials, and the physiological and molecular changes such as chlorophyll content, oxidizing agents such as O_2_^−^ and H_2_O_2_ content, the activities of antioxidant enzymes (POD, SOD, and CAT), and the AsA-GSH cycle (AsA and GSH content, and APX and GR activities) were investigated. Furthermore, we also studied the expression levels of leaf senescence related genes (*SAG12* and *SAG13*) to explore the molecular mechanism of MT on grapevine leaf senescence.

## 2. Materials and Methods

### 2.1. Plant Material and MT Treatment

The experiment was performed at Gansu Province Engineering Research center of grapevine seedling in Wuwei Academy of Forestry Science, Gansu, China. Fully matured healthy leaves were detached with their complete petioles from five-year-old trees of ‘Red Globe’ in the greenhouse on 9 October 2018. Then, all leaves were covered with wet absorbent gauze in the ice box and immediately transferred to the laboratory. Leaves were rinsed with distilled water and soaked in the different concentrations of MT including 50, 100, 200, 500 μM with 0.1% (v/v) Tween-80 for 60 seconds. For each treatment, 50 leaves were selected and dried at room temperature. Finally, each leaf was transferred and incubated in a growth chamber at constant temperature (28 °C) and 80% ~ 90% relative humidity (RH) without light. Leaf samples were collected after 0, 4, 8, 12, 16, and 20 days of each treatment, quickly frozen in liquid nitrogen, and stored at −80 °C, then 0.5 g samples were taken from the three mixed leaves to do following examinations, but not for chlorophyll content and electrolyte leakage measurements, the fresh leaves were used for these two tests. For RNA extraction, leaf samples were collected in control (0 μM) and 100 μM MT treatment at different times including 0, 4 and 8 days after dark-induced senescence. 

### 2.2. Measurement of Chlorophyll Content

To measure the chlorophyll content, leaf samples (0.5 g) were extracted with 50 mL acetone and alcohol (2:1 v/v) solution at different times of MT exposure. Then, the absorbance of chlorophyll extracts was determined at 649 nm and 665 nm by a UV-Visible spectrophotometer (UV759CRT, Yoke, Shanghai, China), and chlorophyll content was calculated according to the method described by Lichtenthaler and Wellburn [65]. 

### 2.3. Determination of MDA Content and Electrolyte Leakage

For the measurement of malondialdehyde (MDA) content, 0.5 g of leaf powder was transferred in a chilled solution that contains 5 mL of Trichloroacetic acid solution (100 g·L^−1^). Each mixture was centrifuged at 10 000 g for 20 min at 4 °C, and the supernatant was kept for measuring the MDA content. The mixture of 2.0 mL supernatant and 2.0 mL of 0.67% Thiobarbituric acid boiled for 20 min. After cooling, the mixture was centrifuged again and the absorbance value of the supernatant was determined at 450 nm, 532 nm, and 600 nm. The MDA content was determined according to the method described by Cao et al. [66] and expressed in mM·g^−1^FW 

Electrolyte leakage was measured by the method of Dionisio-Sese et al. [67]. In order to test electrolyte leakage, fresh leaf samples (0.1 g) were cut into pieces and transferred into the tubes containing 10 mL deionized water. The tubes were placed in a water bath at a constant temperature of 32°C for 120 min. Then, the initial electrical conductivity (R1) was tested by an electrical conductivity meter (DDS-307, Rex, Shanghai, China). Next, the tubes boiled for 20 min, cells completely were killed, and all electrolytes released. When temperature cooled down to 25 °C, the final electrical conductivity (R2) was determined. The electrolyte leakage was expressed following the formula: electrolyte leakage (%) = R1/R2 × 100.

### 2.4. Extraction and Antioxidant Enzymes Assay

To prepare the crude enzyme extraction, leaf powder (0.5 g) was transferred in a chilled extracting solution with 9 mL of 0.1 M sodium phosphate buffer (pH 7.8) containing 0.1 mM EDTA-Na_2_ and 1 % polyvinylpyrrolidone. Each mixture was centrifuged at 12 000 g for 20 min at 4 °C, and the supernatant was kept for measuring the antioxidant enzymes activity. 

SOD activity was measured by the nitro blue tetrazolium (NBT) illumination method [68]. Accordingly, 3.3 mL of reaction mixtures was formed of 1.5 mL of 50 mM sodium phosphate buffer (pH 7.8), 0.3 mL of 130 mM methionine, 0.3 mL of 750 μM NBT, 0.3 mL of 100 μM EDTA-Na_2_, 0.3 mL of 20 μM riboflavin, 0.1 mL of the enzyme extract, and 0.5 mL of distilled water. Then, the color reaction of mixtures was at a light intensity of 4000 lx for 20 min. After the reaction finished, we used the black cloth to terminate the color reaction. Finally, we monitored the SOD activity at 560 nm according to the inhibition of the photochemical reduction of NBT. One unit of SOD activity was defined as the amount of enzyme needed to contain NBT photochemical reduction of 50%. 

The activity of POD was determined at 470 nm by a UV-Visible spectrophotometer [69]. 10.0 mL of reaction mixtures contained 1.0 mL of the enzyme extract, 1.0 mL of 0.1% (m/v) guaiacol, 7.0 mL distilled water, and 1.0 mL 0.18% (v/v) H_2_O_2_. After blending, they reacted for 10 min at 25 °C. This reaction began after adding the H_2_O_2_. The POD activity of the extracts was presented in µg·g^−1^FW·min^−1^.

CAT activity was detected by recording the decrease in absorbance at 240 nm [70], as a result of the decomposition of H_2_O_2._ The reaction mixture (3.0 mL) contained 2.9 mL of 20 mM H_2_O_2_ and 0.1 mL of the enzyme extracts. This reaction was also initiated by adding H_2_O_2._ After 15 seconds of reaction, the absorbance was recorded every 30 seconds. The CAT activity of the enzyme extracts was expressed in U·min^−1^·g^−1^FW.

The activity of APX was measured at 290 nm by the decrease in absorbance because the reduced ascorbate was oxidized [71]. 3.0 mL of reaction mixtures consisted of 2.6 mL of reaction buffer with 0.1 mM EDTA-Na_2_ and 0.5 mM ascorbate, 0.1 mL of enzyme extract, and 0.3 mL of 2 mM H_2_O_2_. This reaction was initiated by adding H_2_O_2_. After 15 seconds of reaction, the absorbance was monitored every 30 seconds. The APX activity of the enzyme extracts was expressed in U·min^−1^·g^−1^FW. 

For the GR activity, it was measured according to the method of Carlberg and Mannervik [72]. 3.0 mL of reaction mixtures consists of 2.7 mL of 0.1 M sodium phosphate buffer (pH 7.5) with 1.0 mM EDTA-Na_2_, 0.1 mL 5.0 mM oxidized glutathione, and 0.2 mL of enzyme extract. This reaction was initiated by adding 0.04 mL 4.0 mM nicotinamide adenine dinucleotide phosphate (NADPH). After 15 seconds of reaction, the absorbance was monitored every 30 seconds. The GR activity of the enzyme extracts was expressed in U·min^−1^·g^−1^FW. 

### 2.5. Extraction and Analysis of Antioxidant Substances

Briefly, 0.5 g of leaf tissues was crashed using liquid nitrogen and transferred into 8 mL cold 5% (m/v) sulfosalicylic acid and blended well. The mixture was centrifuged at 16 000 g for 25 min at 4 °C, and the supernatant was removed for the following tests.

The AsA was measured by the phenanthroline colorimetric method [73]. 5 mL of reaction mixtures included 1.0 mL of supernatant, 1.0 mL of 50 g·L^−1^ (m/v) TCA, 1 mL of absolute ethanol, 0.5 mL of 0.4% (v/v) phosphoric acid-alcohol solution, 1.0 mL of 5 g·L^−1^ (m/v) phenanthroline-alcohol solution, and 0.5 mL of 0.3 g·L^−1^ FeCl_3_-alcohol solution. The mixture was incubated at 30 °C for 60 min. The absorbance of the reaction system was determined at 534 nm. The content of the AsA was expressed in mg·g^−1^FW. GSH was determined according to the methods of Griffith [74]. The 2.5 mL of the reaction system included 1.0 mL of the supernatant, 1.0 mL of 0.1 mM sodium phosphate buffer (pH 7.7), and 0.5 mL 4 mM 5,5’-dithio-bis-2-nitrobenzoic acid (DTNB) (dissolved in potassium phosphate buffer pH 6.8. The reaction was incubated at 25 °C for 10 min. Finally, the absorbance was recorded at 412 nm. The content of GSH was expressed in nM·g^−1^FW.

### 2.6. Quantifications of O_2_^−^ and H_2_O_2_

The O_2_^−^ production rate was measured according to method described by Zhang et al. [75]. Briefly, 0.5 g leaf tissue was ground using liquid nitrogen, homogenized in 5.0 mL 0.05 mM sodium phosphate buffer (pH 7.8) containing 1 mM EDTA, 0.3% Triton X-100, and 2% PVP and centrifuged at 12 000 g for 20 min at 4 °C. Successively, 1.0 mL supernatant, 1.0 mL PBS (pH 7.8), and 1.0 mL 1 mM hydroxylamine hydrochloride were mixed and incubated at 25 °C for 60 min. Then, 1.0 mL 17 mM p-aminobenzene sulfonic acid and 1.0 mL 7.0 mM naphthylamine were added to the PBS and hydroxylamine hydrochloride mixture and incubated at 25°C for 20 min. The absorbance was measured at 530 nm using a spectrophotometer (UV759CRT, Yoke, Shanghai, China). The production rate was expressed in nM·min^−1^·g^−1^FW.

The content of H_2_O_2_ was measured according to the method described by Yahmed et al. [76]. Briefly, 0.5 g leaf tissue was crashed using liquid nitrogen and homogenized in 5.0 mL cold 0.1% (m/v) TCA. The homogenate was centrifuged at 12 000 g for 20 min and 0.5 mL of the supernatant was added to 0.5 mL of 0.1 mM sodium phosphate buffer (pH 7.0) and 1.0 mL 1.0 M of potassium iodide. The mixture was incubated in dark at 28 °C for 15 min. The absorbance was measured at 390 nm. The content of H_2_O_2_ was based on a standard curve generated with known H_2_O_2_ concentrations. 

### 2.7. RNA Isolation and Quantitative Real-time PCR

Total RNA of leaves was extracted with RNA extraction kit (Real-Times Biotechnology, Beijing, China) according to the manufacturer’s instructions. The quality of RNA was examined by 1% (m/v) agarose gel and further assessed by Nanodrop^TM^ 2000 Spectrophotometer (Thermo Fisher, New York, NY, USA).

The qRT-PCR was performed by Real-Time fluorescence quantitative PCR instrument (LightCyclery 96-Real-Time PCR system, Roche, Switzerland) with SYBR Green PCR Master Mix (Takara, Kusatsu, Japan). The thermal profile was used: 95 °C for 15 min, followed by 95 °C for 10 s, 60 °C for 30 seconds, and 72 °C for 30 seconds for 40 cycles. The expression levels of three senescence-associated genes (*SAG12CysProt* and *SAG13*) were analyzed by the comparative 2^−ΔΔCT^ method. The qRT-PCR experiments were performed for three biological replications. The primer sequences of *SAGs* [77] with modifications for qRT-PCR were shown in Table 1.

### 2.8. Statistical Analysis

All data were analyzed by one-way ANOVA method, followed by Duncan’s multiple range tests. The data in all figures were presented as ‘means ± standard deviation (S.D.)’ with three replications.

## 3. Results

### 3.1. MT Could Monitor Dark Yellowing in a Dose-Dependent Behavior

**Figure 1:**
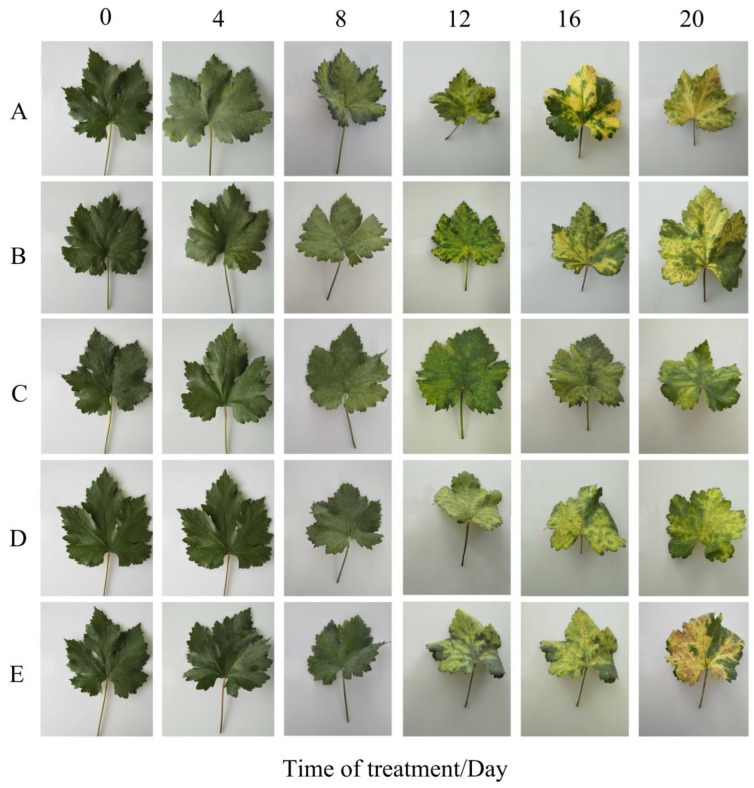
Effects of MT on phenotypical traits of grapevine leaves at different time courses (0, 4, 8, 12, 16, and 20 d) after dark treatment. Grapevine leaves were treated with MT and labeled with (**A**) as control (distilled water), (**B**) 50 μM, **(C**) 100 μM, (**D**) 200 μM, and (**E**) 500 μM MT. All the leaves were incubated in a dark growth chamber where temperature and RH were held at 28 °C and 80%–90% without light, respectively. For each treatment, 50 leaves were considered.

To validate whether exogenous MT application has effect on the dark-induced leaf senescence in grapevine, the different concentrations of MT (0, 50, 100, 200, and 500 μM) were used, and the leaf appearance was investigated in different time courses (0, 4, 8, 12, 16, and 20 d). Since leaf yellowing is one of the most direct senescence symptoms, we observed a difference between samples treated with and without MT. As shown in Figure 1A, the detached leaves of grapevine in the control treatment gradually turn yellow under dark conditions (Figure 1A) from the 8th day, while the leaves with MT treatments were kept healthy at this time point. Color changing continued 12 to 20 days after dark treatment at different concentrations of MT, including 50 μM, 200 μM, and 500 μM (Figure 1B,D,E), and control (Figure 1A) while concentration 100 μM of MT could slow down the dark yellowing until to 20th day (Figure 1C). These results demonstrated that 100 μM of MT treatment could significantly delay the dark-induced leaf senescence.

**Figure 2:**
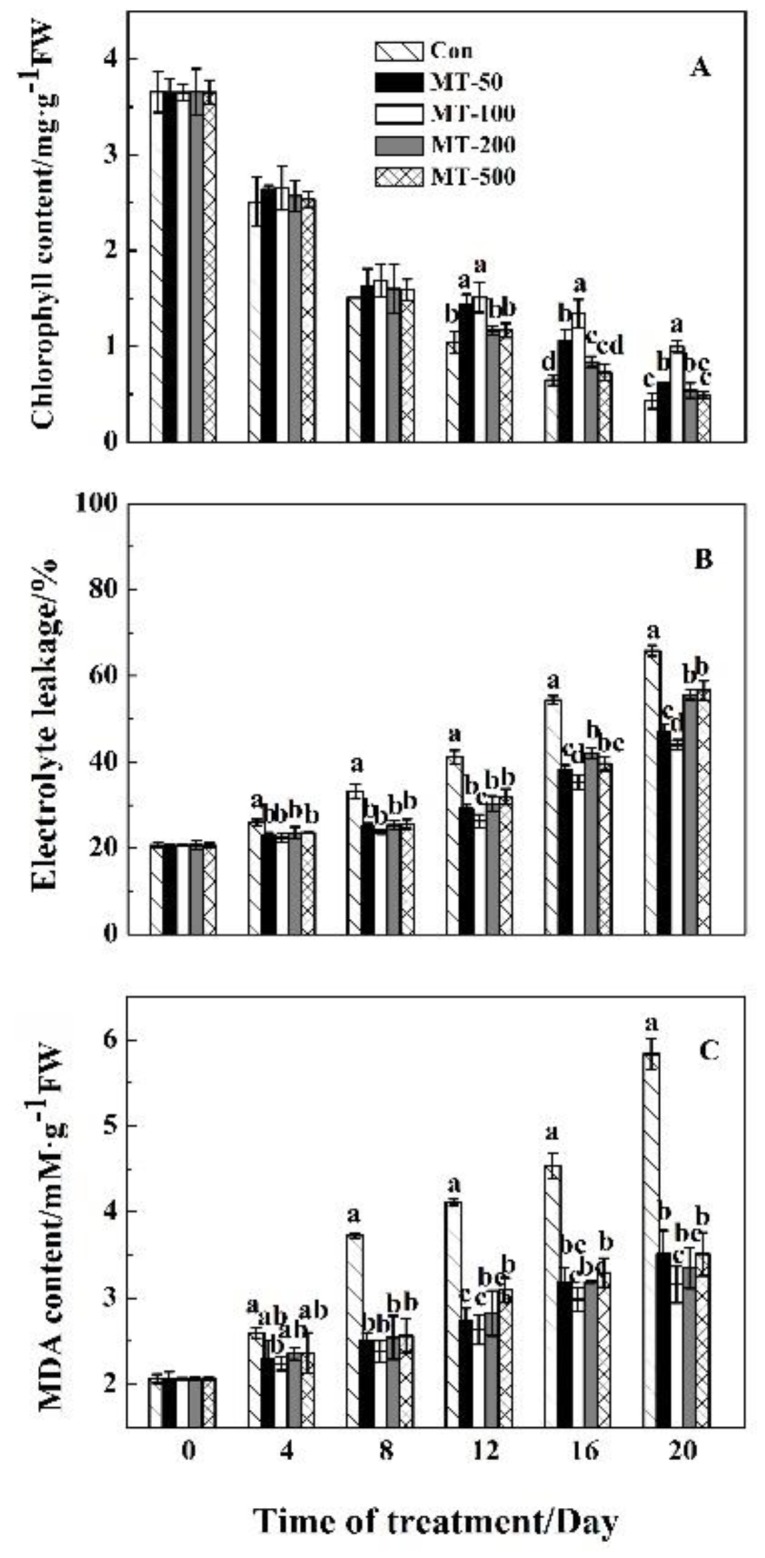
Effects of MT on the contents of chlorophyll (**A**), electrolyte leakage (**B**) and MDA content (**C**) of detached grapevine leaves during dark-induced senescence. Different letter indicates significant differences at the level of 0.05.

Since leaf senescence has a relationship with chlorophyll content, electrolyte leakage, and MDA content, we evaluated these parameters in grapevine leaves treated with MT and compared them to controls. As shown in Figure 2A, although dark condition in grapevine leaves decreased the chlorophyll content in a time-dependent manner, there is no significant difference among the samples treated with and without MT at time points 0, 4, and 8 days after exposure. The changes of chlorophyll content start to appear between different concentrations of MT from the 12th to the 20th day after exposure. For example, on the 12th day, the chlorophyll content with 100 μM pre-treatment was 1.51 mg·g^−1^FW, whereas it was 1.04 mg·g^−1^FW in the control treatment, which was statistically significant. Results also indicated that 100 μM MT treatment could preserve the chlorophyll content at a higher level than the other treatments. 

The electrolyte leakage measurements indicated that MT had different effects on the treated and non-treated samples (Figure 2B). The ion leakage decreased in initial stages at the onset of senescence in grapevine leaves (four days) in response to different concentrations of MT and decrement continued during time series after MT exposure. Among different concentrations of MT, 100 μM concentration could significantly decrease the electrolyte leakage in comparison to other treatments, especially to normal conditions.

Analysis of MDA also exhibited that MT in different concentrations began to decrease MDA content during time series after exposure compared to control. However, MDA content increased with prolonged MT treatment, but the increment was lower in different concentrations of MT than in control treatment from days 4 to 20 (Figure 2C). Among different concentrations of MT, the concentration of 100 μM showed a significant decrease in all-time series after exposure. 

According to obtained results of the chlorophyll content, electrolyte leakage, and MDA content, we observed that 100 μM MT treatment had the best effect on inhibiting the dark induced leaf senescence. Therefore, we preferentially chose this concentration to perform the next analysis. 

### 3.2. MT Could Decline the Accumulation of Oxidizing Agents

**Figure 3:**
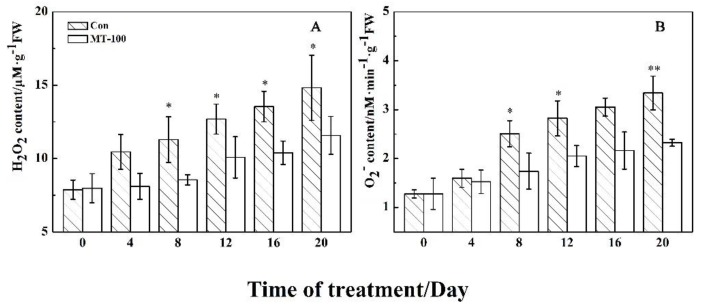
Effects of MT on accumulation of H_2_O_2_ (**A**) and O_2_^−^ (**B**) in detached grapevine leaves during dark-induced senescence. ** and * indicate significant differences at 0.01 and 0.05 levels, respectively.

Since senescence can involve oxidative damage, we examined the effect of MT on the content of reactive oxygen species (ROS) like superoxide (O_2_^−^) and hydrogen peroxide (H_2_O_2_) as strong oxidizing agents in grapevine leaves during dark-induced senescence. As shown in Figure 3A,B, the accumulation of H_2_O_2_ and O_2_^−^ increased gradually during time series in control samples. Although the changes pattern in the content of oxidizing agents was similar, the concentration of H_2_O_2_ and O_2_^−^ were significantly lower after treatment with 100 μM MT during times 8–20 day when compared to the controls, with exception O_2_^−^ concentration at the 16th day (Figure 3B). Since O_2_^−^ can be as substrate in the H_2_O_2_ synthesis reaction, this result suggests that 100 μM MT treatment could significantly suppress H_2_O_2_ and O_2_^−^ production in detached grapevine leaves.

### 3.3. Antioxidant Enzyme Responses to MT Treatment 

**Figure 4:**
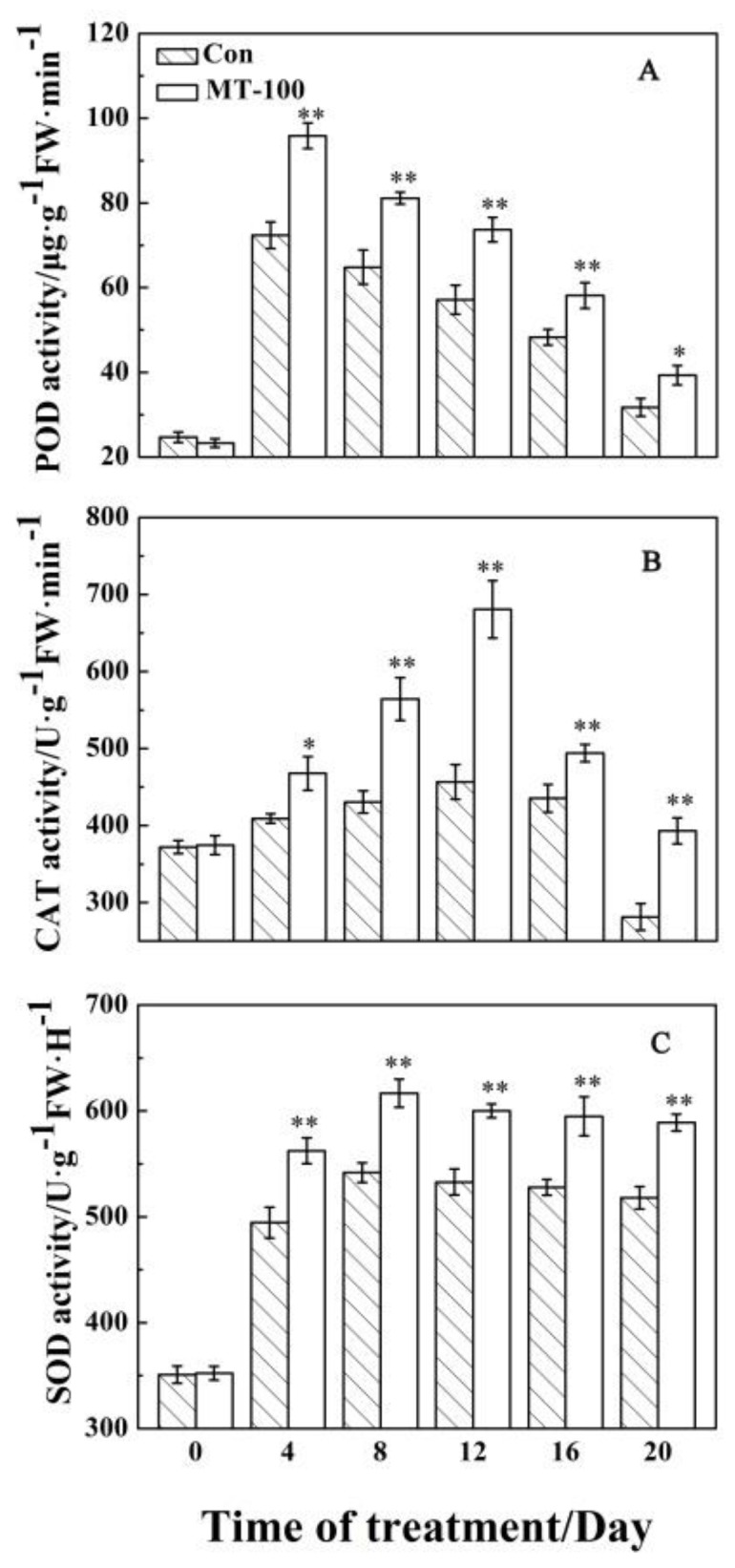
Effects of MT on activity of POD, CAT and SOD in detached grapevine leaves during dark-induced senescence. (**A**) POD activity. (**B**) CAT activity. (**C**) SOD activity. Both of treated and non-treated samples with MT were compared at each time point. ** and * indicate significant differences at levels of 0.01 and 0.05, respectively.

Oxidant agents require the antioxidant system, especially antioxidant enzymes including POD, CAT, and SOD, which can effectively scavenge the reactive oxygen species in a defense system. In order to test whether MT treatment could promote the activity of these enzymes, the activity of POD, CAT, and SOD was evaluated. As shown in Figure 4, the activity of these three antioxidant enzymes changed and showed the similar trends during leaf senescence. With prolonged MT exposure, the activities of POD, CAT, and SOD in the detached leaves indicated the similar patterns of changes that enhanced at first, and then reduced in both treatments. Moreover, the rate of decrement of POD and CAT activity (Figure 4A,B) is greater than that of SOD (Figure 4C). The activity of POD and CAT reached a peak at days 4 and 12, respectively, while SOD showed a high level of activity during days 8 to 12 after MT exposure. Moreover, the MT pretreatment could significantly increase the activities of POD, CAT, and SOD when compared to control treatment (Figure 4). It demonstrated that MT treatment could increase the activity of antioxidant enzymes to scavenge ROS production and protect leaves from senescence.

### 3.4. Effect of MT on the Ascorbate-Glutathione Cycle 

**Figure 5:**
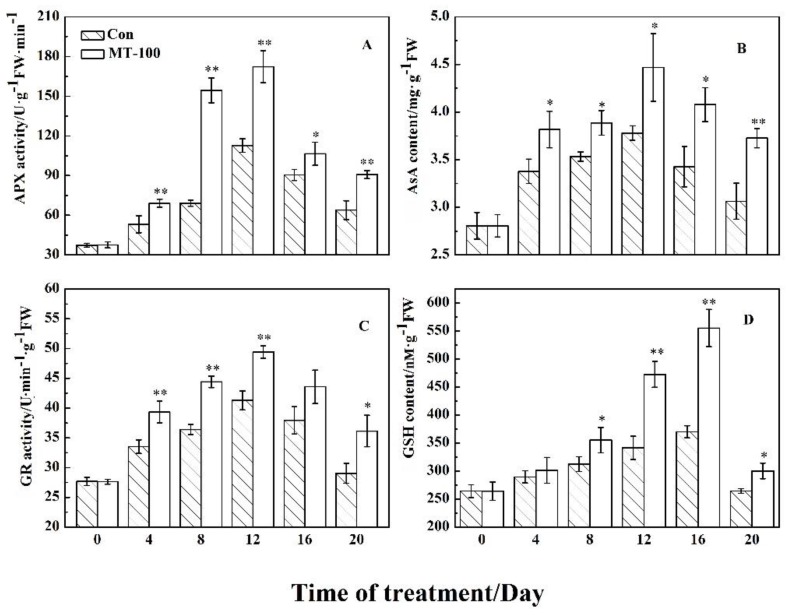
Effects of MT on the content of primary antioxidants and activity of main enzymes involved in AsA-GSH cycle in detached grapevine leaves during dark-induced senescence. (**A**) APX activity. (**B**) AsA content. (**C**) GR activity. (**D**) GSH content. ** and * indicate significant differences at 0.01 and 0.05 levels.

To continue the survival of leaves in senescence condition, a balance between oxidant-antioxidant systems is required. AsA-GSH is an important antioxidant system in plants, which can synergize with other ROS scavenging systems to remove excessive accumulation of oxidizing agents. This cycle involves the key antioxidants compounds including AsA and GSH, and the main enzymes like APX, GR [78,79]. For example, AsA could function directly to detoxify H_2_O_2_. Thus, to investigate the relationship between MT treatment and AsA-GSH cycle, we evaluated the changes of AsA and GSH contents as antioxidant metabolites, and the activities of APX and GR linking these metabolites. As shown in Figure 5, MT treatment significantly increased the content of AsA (during times 4 to 20 day) and GSH (during times 8 to 20 day) in comparison to control treatment (Figure 5B,D). Moreover, MT treatment could significantly hike the activities of APX and GR compared to control treatment. The behavior of the two enzymes was similar during the time series after MT exposure because their activity was raised from days 0 to 12 and then fell from days 12 to 20 (Figure 5A,C). These results elucidated that AsA-GSH cycle plays an important role with MT treatment to delay or suppress leaf senescence.

### 3.5. MT Could Inhibit the Expression Levels of SAGs

**Figure 6:**
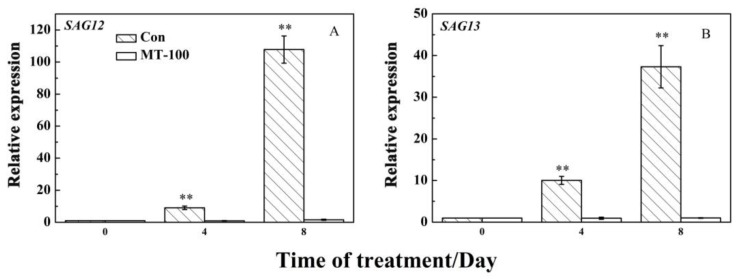
Effects of MT on senescence-associated genes (SAGs) expression in detached grapevine leaves during dark-induced senescence. (**A**) Expression level of *SAG12.* (**B**) Expression level of *SAG13.* ** and * indicate significant differences at 0.01 and 0.05 levels, respectively.

Leaf senescence involves regulatory pathways related to gene expression in the senescence program, especially senescence-associated genes (SAGs). In order to verify that MT treatment could delay or inhibit leaf senescence at the molecular level, we quantified transcript levels of some senescence-associated genes or senescence-up-regulated genes (SAGs) in grapevine leaves. The relative expression levels of two genes *SAG12* and *SAG13* were dramatically upregulated at days 4 and 8 under treatment and reached their maximum levels after 8 days (Figure 6). Meanwhile, pretreatment with 100 μM MT indicated that the levels of gene expression follow a similar trend in response to MT and decrease during days 4 and 8 after exposure (Figure 6). After 8 days, the expression levels of *SAG12* and *SAG13* were *67.75*-folds and 36.73-folds higher in the control than MT treatment, respectively (Figure 6A,B). These results showed that MT treatment could inhibit the expression of SAGs such as *SAG12* and *SAG13*.

## 4. Discussion

Leaf senescence plays an important role in plant life cycle and is mainly affected by low temperature, uncomfortable light, drought, pathogens attack, and hormones. We induced leaf senescence by dark treatment and examined the function of MT on grapevine leaf senescence. Many studies reported that darkness induce senescence when individual leaves are detached, but not when whole plants are darkened [80,81,82]. Therefore, in the current study, the treatment of darkness on detached grapevine leaves was performed. This system could contribute to study mechanisms of leaf senescence on other species. In the senescence program, chloroplast disintegration and chlorophyll degradation happen at the cellular level and leaf color changes from green to yellow, which is the most remarkable phenotype of leaf senescence [83]. Therefore, the change of chlorophyll content is one of the most important and typical indicators to evaluate leaf senescence. MT is involved during plant growth and development. It also could inhibit leaf senescence that has been widely studied in perennial ryegrass [56], kiwifruit [57], apples [58,62], and adzuki bean [84]. In this work, exogenous MT application on detached grapevine leaves could inhibit yellowing in the dark (Figure 1) through slowing down the chlorophyll degradation rate (Figure 2A) that led to delay dark-induced leaf senescence. These results strongly suggested that exogenous application of MT had a positive effect on delaying grapevine leaf senescence.

During leaf senescence, reactive oxygen species (ROS) are produced and the activities of antioxidant enzymes including CAT, SOD and POD decrease, which resulted in the imbalance of ROS metabolism [8,85]. In addition, excessive ROS could oxidize cell membrane lipid, which directly destroys the biological membrane system and leads to electrolyte leakage and MDA production. In this study, we found that electrolyte leakage (Figure 2B), O_2_^−^ concentrations (Figure 3B), MDA and H_2_O_2_ content (Figure 2C and Figure 3A) continuously increased during leaf senescence, while pre-treatment of MT on detached grapevine leaves reduced the production of MDA (Figure 2C), H_2_O_2_ content (Figure 3A), and O_2_^−^ concentrations (Figure 3B), and also inhibits electrolyte leakage (Figure 2B). This indicated that MT could modulate the production of ROS to slow down the dark-induced leaf senescence. Moreover, MT was an antioxidant substance that could directly reduce ROS level in organisms. Consequently, it would alleviate damage to the membrane system [86,87,88]. In addition, some researchers suggested that MT treatments could also improve the activities of SOD, CAT and POD in the process of leaf senescence [56,57,58,62]. This is consistent with our study that the three enzymes activities increased firstly and then decreased with the aging process within MT treatment (Figure 4). Overall, MT could scavenge of ROS by activating the antioxidant enzymes to delay grapevine leaf senescence. 

In the AsA-GSH cycle system, APX and GR are two key enzymes, which eliminate ROS accompanied with SOD, POD, and CAT enzymes to maintain the balance between oxidant (ROS) and antioxidant systems and keep the stability of cell membrane [89,90]. APX is a key enzyme in chloroplast to detoxify H_2_O_2_ through AsA. GR is one of the pivotal enzymes to maintain the effective function of AsA-GSH cycle through reducing oxidized glutathione (GSSG) to GSH by an NADPH-dependent pathway [91,92]. Here, the AsA and GSH concentrations increased (Figure 5B and D) with the reduced production of O_2_^−^ and H_2_O_2_ (Figure 3), meanwhile, the APX and GR activities were enhanced (Figure 5A,C) during grapevine leaf senescence. Furthermore, AsA and GSH contents, and the activities of APX and GR (Figure 5) were significantly higher in the treated samples with MT than in the controls, demonstrating that MT could modulate the AsA-GSH cycle system to remove ROS. This was consistent with the studies on apples and ryegrass in which exogenous MT treatment could regulate the AsA-GSH cycle to delay leaves senescence with the enhanced APX and GR activities as scavengers of ROS, as well as the higher concentration of AsA [56,58]. 

Moreover, during the senescence program, most of the genes involved in leaf senescence were upregulated, such as SAG12 and SAG13 [11,93]. SAG12 in *Arabidopsis*, a gene encoding a cysteine protease, is highly specific in aging and is often used as a marker gene for aging [94]. A previous study showed that SAG12 was definitely activated by developmental senescence, but not triggered by the regulation of hormone or in response to stresses [95]. For example, during natural senescence in grapevine, the SAG12 also was activated [77]. In addition, PeSAG12-1 was highly induced with the increase of ROS production during dark-induced senescence of *Pelargonium* cuttings [96]. Furthermore, the expression of many other SAGs was also induced by ROS [97,98], indicating that there is a close relationship between ROS and SAG genes during leaf senescence. Present results indicate that pretreatment of MT dramatically suppressed SAGs expression at 8 days, while the controls had higher expression levels (Figure 6). Therefore, MT could repress the expression of SAG genes to delay grapevine leaf senescence.

**Figure 7:**
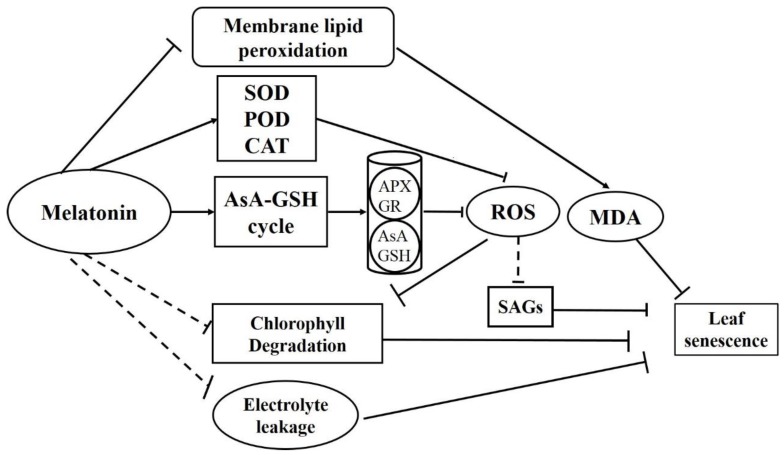
The model of MT treatment on the effect of dark-induced grapevine leaf senescence. The broken lines represent the unknown molecular mechanism.

According to the above discussion, we came to a model where the dark induced grapevine leaf senescence triggered the high production of ROS, which could further activate the expression of SAGs (Figure 7). Pre-treatment of MT could activate the antioxidant enzymes and AsA-GSH cycle system to reduce the production of ROS, which finally prevent the expression of SAGs to delay dark induced grapevine leaf senescence (Figure 7). 

## 5. Conclusions

Senescence or biological aging is a time-dependent process. During darkness, pre-treatment of MT could significantly decrease the production of ROS and increase the activity of antioxidant enzymes including SOD, CAT, and POD. In addition, MT activates the AsA-GSH cycle system during senescence program, raises the content of AsA and GSH, and induces activities of APX and GR. Meanwhile, MT treatment could suppress the expression of leaf senescence related genes (SAGs). These results suggest that MT can be involved in cellular homeostasis to preserve a balance between oxidant-antioxidant systems that led to a delay in the dark-induced leaf senescence. 

## Figures and Tables

**Table 1:** The primer sequences of *SAGs* for qRT-PCR.

Gene Name	Accession Number	Forward Primer Sequence(5′–3′)	Reverse Primer Sequence(5′–3′)
SAG12-Vv	XM002284937.3	TGAAGGATGCAATGGGGGAC	TCTGCCATCGGAAGCTTTGT
SAG13-Vv	XM002282719.4	TCCTACAAGTGTTTGTGAACGC	ATAGTGGAGCCATCCCCTGA
Ubiquitin-Vv	XM003634272.3	GCTCGCTGTTTTGCAGTTCTAC	AACATAGGTGAGGCCGCACTT
